# Response to “Diagnostic Caveats and Clinical Pitfalls in the Overlap of Leprosy and Para-Kala-Azar Dermal Leishmaniasis”

**DOI:** 10.4269/ajtmh.26-0451a

**Published:** 2026-08-18

**Authors:** Rowena Lee Ying Kwan, Hui Yi Chia, Shawn Vasoo, Jiun Yit Pan

**Affiliations:** National University of Singapore Yong Loo Lin School of Medicine Singapore E-mail: rowenakwan@u.nus.edu; National Skin Centre Singapore E-mail: hychia@nhghealth.com.sg; National Centre for Infectious Diseases Singapore E-mail: shawn.vasoo@nhghealth.com.sg; National Skin Centre Singapore E-mail: jypan@nhghealth.com.sg

Dear Editor,

We thank Kumar et al.^1^ for their interest in our article, “Post-Kala-Azar Dermal and Visceral Leishmaniasis in a Patient from Bangladesh”^2^ and appreciate the opportunity to respond to their concerns regarding our diagnostic approach and treatment choice.

Firstly, regarding the baseline evaluation for leprosy, the patient did not exhibit neurological deficits. We thank the authors for highlighting this point.

Secondly, concerning the initial therapeutic choice, the working diagnosis and treatment were for multibacillary leprosy in reaction, in view of his clinical presentation of extensive infiltrated plaques with a positive slit-skin smear (SSS). Accordingly, the initial management with World Health Organization multidrug therapy (WHO-MDT) and prednisolone was appropriate at the time.

Lastly, regarding the histological images and the question of systemic involvement, while the patient exhibited hepatosplenomegaly, other classic hallmarks of visceral leishmaniasis, such as progressive weight loss and severe anemia (he presented with moderate anemia with Hb 9.3–10.6 g/dL, secondary to WHO-MDT related hemolysis), were not present. Despite the slit-skin smear positivity, the Ziehl-Neelsen and Fite stains performed on the histology specimen were negative. It was precisely this discordance between the SSS and skin biopsy findings that prompted our re-evaluation of the histology. The *Leishmania* amastigotes were subsequently identified on hematoxylin and eosin staining, and CD1a immunostain (clone MTB1) was also performed, and this stain highlighted the organisms. Ophthalmological evaluation of the patient revealed conjunctivitis, with no evidence of uveitis, glaucoma, or cataracts. We were unfortunately unable to pursue further testing on repeat samples (such as molecular testing) for leishmaniasis, as the patient had returned to his home country.

The patient was subsequently informed of histological findings via telephone and advised to seek evaluation from local infectious diseases physicians.

We hope that these details clarify the diagnostic journey and management decisions in this challenging case of para-kala-azar dermal leishmaniasis mimicking leprosy.

Sincerely,
